# Chimerism through the activation of invariant natural killer T cells prolongs graft survival after transplantation of induced pluripotent stem cell–derived allogeneic cardiomyocytes

**DOI:** 10.1371/journal.pone.0264317

**Published:** 2022-03-02

**Authors:** Shohei Yoshida, Shigeru Miyagawa, Takashi Matsuzaki, Yasuyuki Ishii, Emi Fukuda-Kawaguchi, Takuji Kawamura, Ai Kawamura, Yuki Nakamura, Koichi Toda, Yoshiki Sawa

**Affiliations:** 1 Department of Cardiovascular Surgery, Osaka University Graduate School of Medicine, Osaka, Japan; 2 Department of DDS Pharmaceutical Development, Osaka University Graduate School of Medicine, Osaka, Japan; 3 REGiMMUNE Corp, Tokyo, Japan; 4 Department of Immunological Diagnosis, Juntendo University Graduate School of Medicine, Bunkyo City, Japan; University of Toledo, UNITED STATES

## Abstract

The loss of functional cells through immunological rejection after transplantation reduces the efficacy of regenerative therapies for cardiac failure that use allogeneic induced pluripotent stem cell-derived cardiomyocytes (iPSC-CMs). Recently, mixed-chimera mice with donor-specific immunotolerance have been established using the RGI-2001 (liposomal formulation of α-galactosyl ceramide) ligand, which activates invariant natural killer T (iNKT) cells. The present study aimed to investigate whether mixed chimerism, established using RGI-2001, prolongs graft survival in allogeneic iPSC-CM transplantation. Mixed-chimera mice were established via combinatorial treatment with RGI-2001 and anti-CD154 antibodies in an irradiated murine bone marrow transplant model. Luciferase-expressing allogeneic iPSC-CMs were transplanted into mixed-chimera and untreated mice, followed by *in vivo* imaging. RGI-2001 enhanced iNKT cell activation in mice, and mixed chimerism was successfully established. *In vivo* imaging revealed that while the allografts were completely obliterated within 2 weeks when transplanted to untreated mice, their survivals were not affected in the mixed-chimera mice. Furthermore, numerous CD3+ cells infiltrated allografts in untreated mice, but fewer CD3+ cells were present in mixed-chimera mice. We conclude that mixed-chimera mice established using RGI-2001 showed prolonged graft survival after allogeneic iPSC-CM transplantation. This donor-specific immunotolerance might increase the efficacy of regenerative therapies for heart failure with allogeneic iPSC-CMs.

## Introduction

Despite the development of therapeutic options, including left ventricular assist devices and heart transplantation, severe heart failure afflicts many people because of its inherent complications and the shortage of prospective donors [1, 2]. Regenerative medicine using allogeneic induced pluripotent stem cell (iPSC)-derived cardiomyocytes (iPSC-CMs) is being developed as an alternative treatment option [3–5]. Previously reported therapies using allogeneic iPSC-CMs showed that recipient hearts benefit mainly from the paracrine effect caused by the cytokines from the transplanted allogeneic iPSC-CMs. However, to achieve the ultimate goal of cardiomyogenesis, engraftment of a large number of allogeneic iPSC-CMs at the transplanted site is required. Therefore, it is necessary to overcome the immune rejection that occurs after allogeneic iPSC-CM transplantation [6, 7]. Combinatorial treatment with RGI-2001 (liposomal formulation of α-galactosyl ceramide), the ligand activating invariant natural killer T (iNKT) cells, and an anti-CD154 antibody in a sub-lethally irradiated murine bone marrow transplant model reportedly facilitates the establishment of mixed hematopoietic chimerism through the expansion of *in vivo* regulatory T cells (Tregs) [8–10]. Cardiac allografts were subsequently accepted by mixed-chimera mice in a donor-specific manner without immunosuppressive agents [11, 12]. Therefore, we hypothesized that allogeneic iPSC-CM transplants could be engrafted in mixed-chimera mice without the need for immunosuppressive agents. The present study aimed to investigate whether mixed chimerism, established through the method described, reduces immune rejection after allogeneic iPSC-CM transplantation in mice.

## Materials and methods

### The mouse model

Seven-week-old BALB/c (H-2K^d^) and C57BL/6 (H-2K^b^) mice were purchased from CLEA Japan (Tokyo, Japan). Animal care procedures were carried out following the “Guide for the Care and Use of Laboratory Animals” (National Institutes of Health publication). Experimental protocols were approved by the Ethics Review Committee for Animal Experimentation of Osaka University Graduate School of Medicine (reference no. 25-045-053). Mice were humanely euthanized by intravenous potassium chloride injection under full anesthesia after the required experiments.

### RGI-2001

RGI-2001 (liposomal formulation of α-galactosyl ceramide: NCT04014790) was prepared in collaboration with REGiMMUNE (Tokyo, Japan).

### Activation of iNKT cells

In total, 10 μg/kg RGI-2001 was intravenously administered to BALB/c (H-2K^d^) mice, and flow cytometry was performed on murine splenocytes to examine iNKT cell activation.

### Induction of hematopoietic chimerism

Mixed chimerism was established in BALB/c mice, as previously reported [12, 13]. Briefly, BALB/c mice received 3 Gy of non-myeloablative whole-body irradiation 3–4 h before the intravenous administration of 2.0 × 10^7^ whole bone marrow cells derived from fully allogeneic C57BL/6 (H-2K^b^) mice. Thereafter, the recipient BALB/c mice were treated with a combination of intravenous 10 μg/kg RGI-2001 and intraperitoneal 0.5 mg anti-CD154 antibody (MR1; BioXcell, West Lebanon, NH, USA).

### Flow cytometry

Splenocytes were harvested from each mouse, and peripheral blood mononuclear cells (PBMCs) were harvested from the whole blood of four mice in each group. Thereafter, the cells were incubated with a mixture of anti-CD3 antibody (100235; BioLegend, San Diego, CA, USA), anti-CD1d tetramer (TS-MCD-1; MBL, Nagoya, Japan), and aqueous α-GalCer (Funakoshi Co., Ltd., Tokyo, Japan), or a mixture of anti-H-2K^b^ antibody (116518; BioLegend) and anti-H-2K^d^ antibody (116606; BioLegend) for 30 min. After incubation with the secondary antibody at ambient temperature for 30 min, the labeled cells were assayed using a FACS Canto II system (Becton Dickinson, Franklin Lakes, NJ, USA).

### Cardiomyogenic differentiation of murine iPSCs *in vitro*

Luciferase-murine iPSCs (959A2-1-6), generated from C57BL/6 murine embryonic fibroblasts, were cultured in the absence of serum and feeder cells, using ESGRO Complete PLUS Clonal Grade Medium (MilliporeSigma, Burlington, MA, USA). The cardiomyogenic differentiation of iPSCs was carried out as previously described, with slight modifications, followed by purification with glucose-free medium supplemented with lactic acid. Then, iPSCs (3 × 10^3^ cells) were resuspended in 100-μL aliquots of differentiation medium [DM; Dulbecco’s modified Eagle’s medium (DMEM; Nacalai Tesque, Kyoto, Japan) supplemented with 15% fetal bovine serum (Biofill, Melbourne, VIC, Australia), 100 mmol/L non-essential amino acids (NEAA; Thermo Fisher Scientific, Waltham, MA, USA), 2 mmol/L l-glutamine (Thermo Fisher Scientific), and 0.1 mmol/L 2-mercaptoethanol (Thermo Fisher Scientific)] containing 0.2 mmol/L 6-bromoindirubin-3′-oxime (BIO; a glycogen synthase kinase-3β inhibitor, to activate the Wnt signaling pathway) (MilliporeSigma), and cultured in 96-well Corning Costar Ultra-Low attachment multiwell plates (MilliporeSigma) for 3 days. On day 3, an additional 100 μL DM without BIO was added to each well. On day 5, individual embryoid bodies (EBs) were transferred to 100-mm gelatin-coated dishes (250 EBs per dish). On days 6, 7, 10, 11, 14, and 15, the medium was exchanged for serum-free modified Eagle’s medium (MEM; Thermo Fisher Scientific) supplemented with insulin transferrin selenium X (Thermo Fisher Scientific). On days 8, 9, 12, and 13, the medium was exchanged with glucose-free DMEM (no glucose, no pyruvate; Thermo Fisher Scientific) supplemented with 4 mmol/L lactic acid (FUJIFILM Wako Pure Chemical Corporation, Osaka, Japan) to purify cardiomyocytes. On day 16, the contracting cell clusters were dissociated, seeded on thermoresponsive 6-well plates (5 × 10^6^ CMs/well; Upcell; CellSeed, Tokyo, Japan), and incubated at 37°C for 2 days. They were then incubated at ambient temperature until the cells spontaneously detached to form scaffold-free cell sheets [14, 15].

### Cell-sheet transplantation

Luciferase transgenic iPSC-CM sheets were subcutaneously transplanted into the dorsa of untreated normal BALB/c mice or mixed-chimera BALB/c mice under general anesthesia via isoflurane inhalation 2 weeks after the establishment of mixed-chimera mice.

### *In vivo* imaging system

Bioluminescent images were acquired using an *in vivo* imaging system (Perkin Elmer, Waltham, MA, USA) on post-operative days 1, 7, 14, (untreated normal BALB/c mice: n = 16, mixed-chimera BALB/c mice: n = 24) and 21 (untreated normal BALB/c mice: n = 8, mixed-chimera BALB/c mice: n = 9). Animals were anesthetized with 2% isoflurane gas in oxygen, and 150 mg/kg d-luciferin (Summit Pharmaceuticals International Corporation, Tokyo, Japan) was intraperitoneally administered. Images were acquired 10 min after injection at the peak of the bioluminescence signal. Images were quantified based on regions of interest over the transplanted region, with data expressed as photon flux (p/s). Percentages of bioluminescent signals relative to those measured 1 day after transplantation were determined to assess graft survival.

To verify the positive correlation between the number of iPSC-CMs and luminescence intensity, we incubated iPSC-CMs for 24 h in 96-well plates containing DMEM and 15% fetal bovine serum and assayed them using a luciferase assay system (Promega Corporation, Madison, WI, USA) on a Synergy HT microplate reader equipped with an injector and with time-resolved fluorescence capability (BioTek Instruments, Inc., Winooski, VT, USA).

### Immunohistochemistry

Dissociated single cells were fixed with 4% paraformaldehyde and labeled with primary anti-alpha-actinin antibody (A7732; MilliporeSigma), followed by incubation with fluorescence-conjugated secondary antibodies, and counterstained with 4′,6-diamidino-2-phenylindole (DAPI; Vector Laboratories, Burlingame, CA, USA). Subcutaneous tissues were collected at day 7 and 21 after transplantation. Harvested tissues around the transplanted site were fixed with 10% buffered formalin, embedded in paraffin, sectioned, and labeled with primary anti-CD3 antibody (B355.1(RIV-9); Abcam, Cambridge, UK), followed by color development with 3,3′-diaminobenzidine. Harvested tissue were also utilized for hematoxylin and eosin staining. The images of multiple locations in the transplant site were taken using a Biorevo BZ-9000 (Keyence, Osaka, Japan). The percentages of stained area were analyzed by ImageJ (National Institutes of Health, Bethesda, MD, USA).

### Statistical analysis

Data were presented as mean ± standard error for continuous variables. Continuous variables were examined using the unpaired Student’s *t*-test. Spearman’s rank correlation was used to compare the relationship between the cell number and luminescence intensity. Statistical analyses were performed using JMP®13 (SAS Institute Inc., Cary, NC, USA). Statistical significance was defined as *P* < 0.05.

## Results

### Effect of RGI-2001 administration

**Fig 1:**
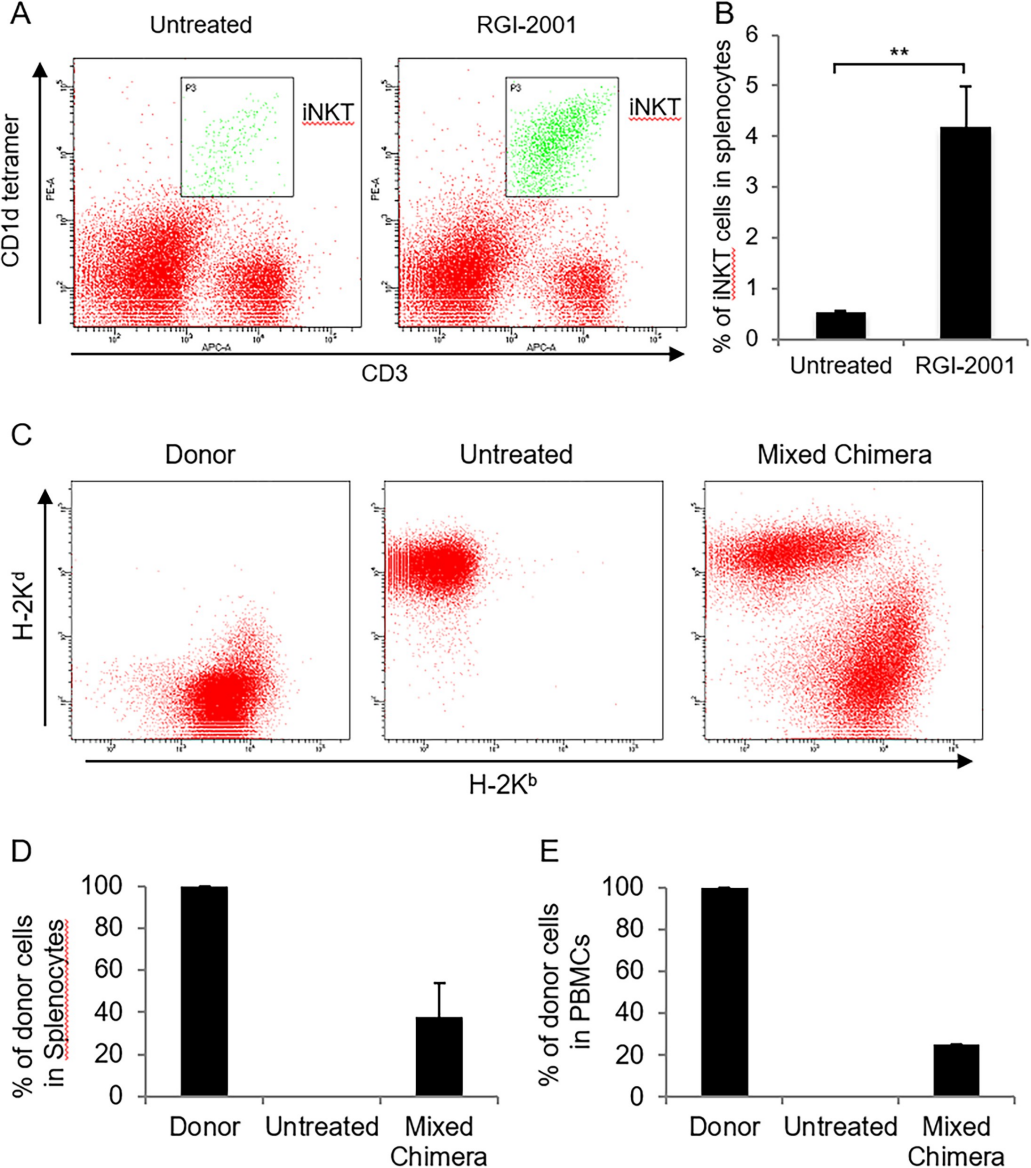
Flow cytometry. (A) Splenocytes were harvested from normal untreated mice or mice intravenously administered with RGI-2001 (liposomal formulation of α-galactosyl ceramide), followed by flow cytometry with a mixture of anti-CD3 antibodies, anti-CD1d tetramer, and aqueous α-GalCer. Representative data in each group are shown. (B) The percentages of invariant natural killer T (iNKT) cells in the splenocytes of mice intravenously administered with RGI-2001 versus the normal untreated mice are shown. (C) Splenocytes and peripheral blood mononuclear cells (PBMCs) were harvested from donor C57BL/6 mice, normal untreated BALB/c mice, or mixed-chimera BALB/c mice and analyzed using flow cytometry with a mixture of anti-H-2K^d^ and anti-H-2K^b^ antibodies. Representative splenocyte data in each group are shown. (D) The percentages of H-2K^b^-positive donor splenocytes of donor C57BL/6, untreated BALB/c, and mixed-chimera BALB/c mice. (E) The average percentages of H-2K^b^-positive PBMCs of donor C57BL/6 mice, normal untreated BALB/c mice, and mixed-chimera BALB/c mice. Student’s *t*-test was performed, and the results are depicted as mean ± SEM. **P < 0.01.

RGI-2001 was intravenously administered at 10 μg/kg to BALB/c (H-2K^d^) mice, and flow cytometry of murine splenocytes was performed to examine iNKT cell activation. The proportion of CD3^+^ and CD1d tetramer-positive cells, indicating iNKT cells, in the splenocytes of BALB/c mice (4.2% ± 0.8%) was increased upon intravenous administration of RGI-2001 compared to that in untreated normal BALB/c mice (0.5% ± 0.0%, *P* = 0.0034) (Fig 1A and 1B).

### Analysis of cells harvested from mixed-chimera mice

Mixed-chimera BALB/c mice were induced as previously reported [12, 13]. Splenocytes and PBMCs were harvested and examined via flow cytometry using anti-H-2K^b^ and anti-H-2K^d^ antibodies for chimerism analysis. Flow cytometry revealed that after 2 weeks, mixed-chimera BALB/c mice harbored both H-2K^d^-positive cells and H-2K^b^-positive splenocytes (H-2K^d^; 62% ± 16%, H-2K^b^; 38% ± 16%) (Fig 1C), as well as PBMCs (H-2K^d^; 75%, H-2K^b^; 25%), while untreated BALB/c mice or donor C57BL/6 mice only harbored H-2K^d^- or H-2K^b^-positive cells, respectively (Fig 1D and 1E).

### Bioluminescence analysis resulted in extended graft survival in mixed-chimera mice

**Fig 2:**
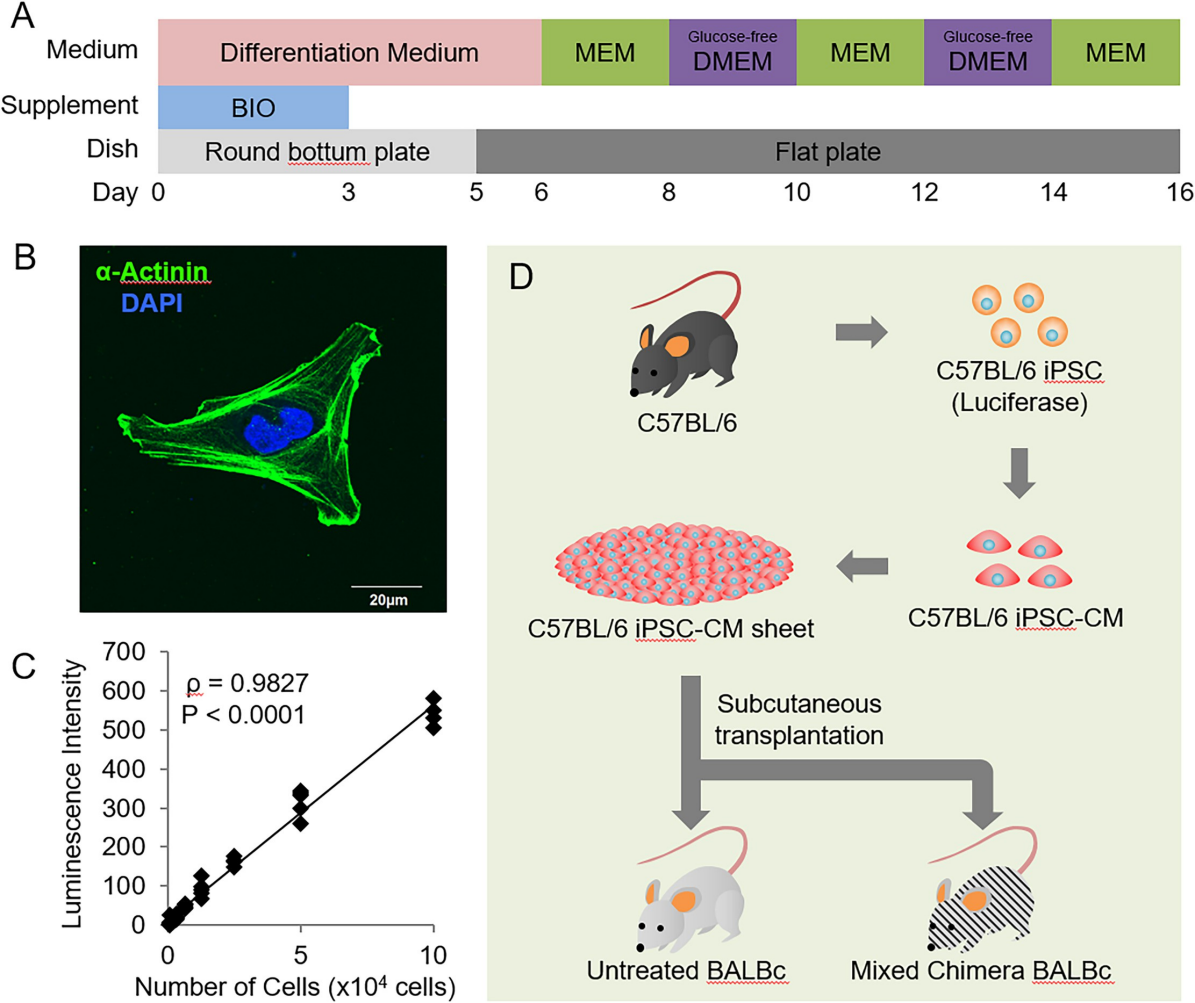
Experimental flow. (A) Cardiomyogenic differentiation protocol. (B) Immunocytochemistry for alpha-actinin (green) and DAPI (blue) in induced pluripotent stem cell (iPSC)-derived cardiomyocytes (iPSC-CMs). Scale bar, 20 μm. (C) Positive correlation between the number of iPSC-CMs and bioluminescence (ρ = 0.9827, *P* < 0.0001). (D) Transplantation scheme of luciferase-expressing murine iPSC-CM sheets derived from C57BL/6 mice into untreated normal BALB/c or mixed-chimera BALB/c mice.

Luciferase-expressing murine iPSCs (959A2-1-6) generated from C57BL/6 murine embryonic fibroblasts were cultured, and murine iPSCs were subjected to cardiomyogenic differentiation. Cell sheets comprising 3.0 × 10^6^ iPSC-CMs/sheet were prepared as previously described (Fig 2A) [14, 15]. The differentiated cardiomyocytes were positive for alpha-actinin (Fig 2B), and *in vitro* bioluminescence imaging analysis of iPSC-CMs revealed a linear relationship between the cell number and the bioluminescence signal (ρ = 0.9827, *P* < 0.0001), validating the use of this method to quantify luciferase-expressing iPSC-CMs (Fig 2C). Luciferase transgenic iPSC-CM sheets were subcutaneously transplanted into the dorsa of untreated normal BALB/c mice or mixed-chimera BALB/c mice 2 weeks after the establishment of mixed-chimera mice (Fig 2D).

**Fig 3:**
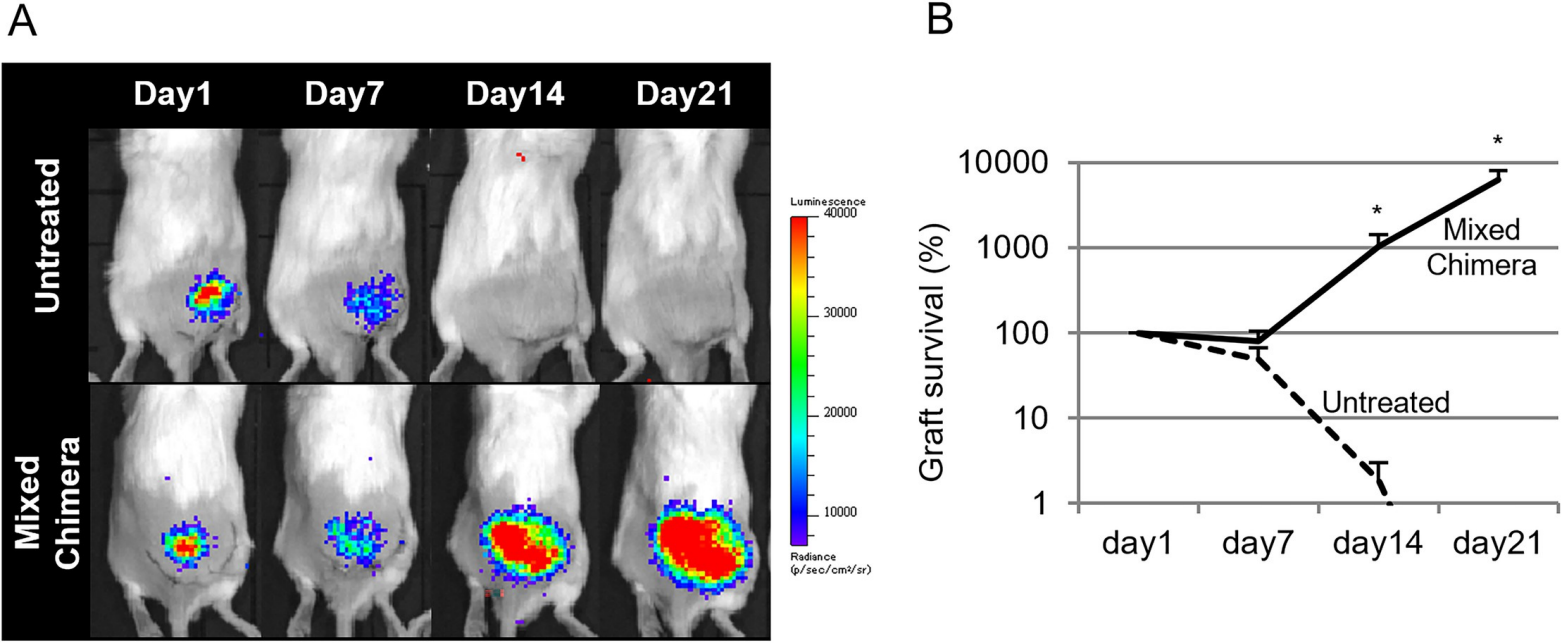
In vivo imaging of bioluminescence. (A) Representative images of luciferase transgenic allogeneic induced pluripotent stem cell-derived cardiomyocyte (iPSC-CM) sheets transplanted in normal untreated BALB/c mice or mixed-chimera BALB/c mice, obtained through *in vivo* imaging on days 1, 7, 14, and 21 post transplantation. (B) Percentages of the bioluminescence signal on days 7, 14, and 21 compared to those on day 1 post transplantation in normal untreated BALB/c mice or mixed-chimera BALB/c mice. Student’s *t*-test was performed, and the results are presented as mean ± SEM. *P < 0.05.

Bioluminescent images were acquired using an *in vivo* imaging system (Perkin Elmer) with intraperitoneal injection of 150 mg/kg d-luciferin (Summit Pharmaceuticals International Corporation) on post-operative days 1, 7, 14, and 21 (Fig 3A). Percentages of bioluminescent signals relative to those on day 1 post transplantation were determined to assess graft survival. *In vivo* imaging revealed that the grafts were obliterated 2 weeks post transplantation in the untreated BALB/c mice (day 7; 48% ± 19%, day 14; 2% ± 1%, day 21; 0% ± 0%); however, grafts were retained for ≥3 weeks in mixed-chimera BALB/c mice (day 7; 81% ± 23%, *P* = 0.3382, day 14; 1029% ± 380%, *P* = 0.0292, day 21; 6210% ± 1831%, *P* = 0.0433) (Fig 3B), indicating that mixed-chimera induction suppresses immune rejection in allogeneic transplantation.

### Histological analysis demonstrated a lower number of CD3^+^ cells in mixed-chimera mice

**Fig 4:**
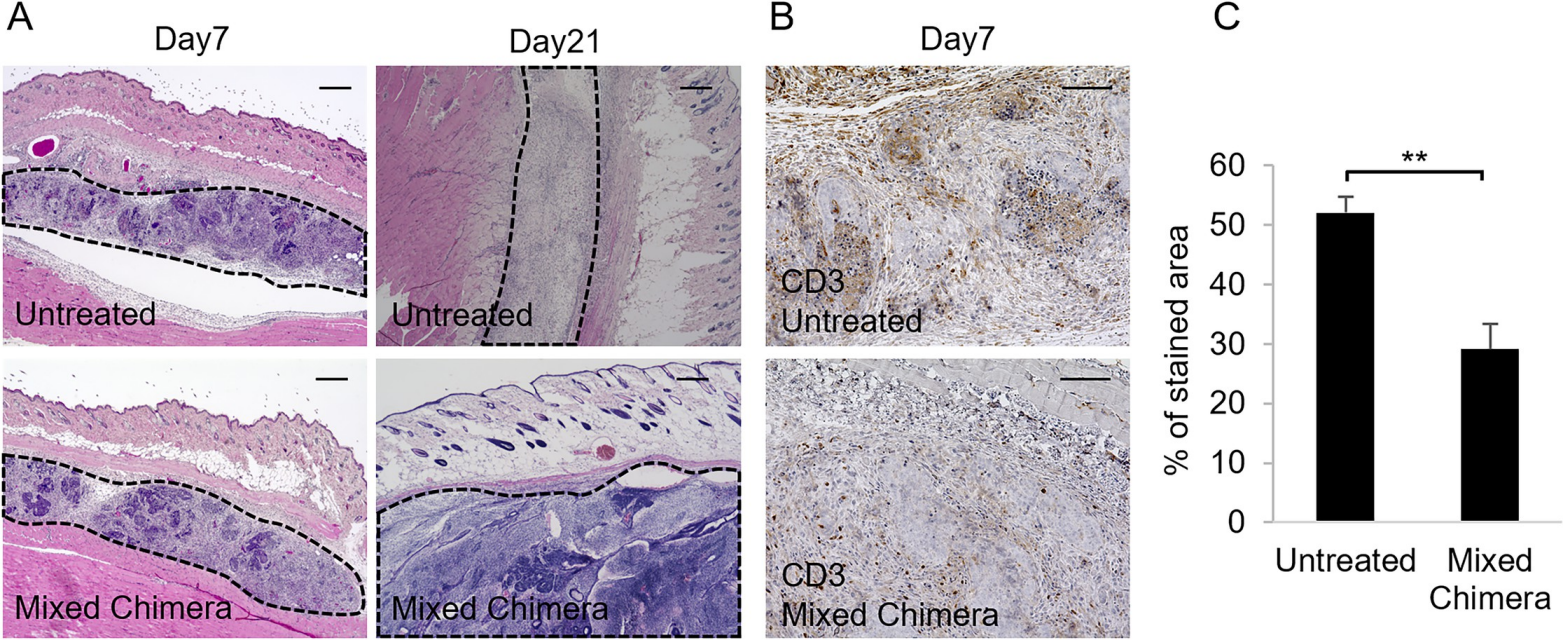
Histochemical and immunohistochemical staining of tissue sections. (A) Representative images showing hematoxylin and eosin staining of tissue sections from normal untreated BALB/c mice (*top*) or mixed-chimera BALB/c mice (*bottom*) 7 days (*left*) and 21 days (*rignt*) post transplantation. Dotted lines indicate surviving induced pluripotent stem cell-derived cardiomyocyte (iPSC-CM) sheets or transplanted site. Scale bar, 300 μm. (B) Representative immunohistochemical staining images using an anti-CD3 antibody for normal untreated BALB/c mice (*top*) or mixed-chimera BALB/c mice (*bottom*) 7 days post transplantation. Scale bar: 100 μm. (C) The percentages of stained area for CD3 in untreated BALB/c and mixed-chimera BALB/c mice. Student’s *t*-test was performed, and the results are depicted as mean ± SEM. **P < 0.01.

To assess the immunological rejection of transplanted iPSC-CMs, we histologically evaluated subcutaneously transplanted iPSC-CMs on days 7 and 21 post transplantation. With hematoxylin and eosin staining, the transplanted sheets were observed to begin to form teratomas in subcutaneous tissues in both groups on day 7. However, larger teratomas formation was observed in mixed-chimera BALB/c mice but not in untreated BALB/c mice on day 21, consistent with the results of bioluminescence analysis (Fig 4A). On day 7, numerous CD3^+^ cells were observed at the site of transplantation in the untreated BALB/c mice, while mixed-chimera BALB/c mice displayed a lower number of CD3^+^ cells at the site of transplantation (untreated 52 ± 3%, mixed chimera 29 ± 4%, *P* = 0.0039) (Fig 4B and 4C), indicating the suppression of immunoreactivity against the transplanted iPSC-CMs in mixed-chimera BALB/c mice.

## Discussion

To induce sufficient cardiomyogenesis using allogeneic iPSC-CMs, overcoming the immune rejection that can occur after allogeneic iPSC-CM transplantation is one of the most important issues. We successfully generated luciferase-expressing iPSC-CMs and mixed-chimera mice and then tested graft survival after the subcutaneous transplantation of allogeneic iPSC-CMs. The survival rate of the transplanted allogeneic iPSC-CMs in the mixed-chimera mice was not reduced even 3 weeks after transplantation, although the grafts were completely eliminated 2 weeks after transplantation in the untreated normal mice. In addition, the immunoreaction with CD3^+^ cells at the site of transplantation decreased in the mixed-chimera mice compared to the untreated mice.

Although major histocompatibility complex (MHC)-matching allogeneic iPSC-CMs transplantation on the surface of an ischemic heart reportedly reduced immune rejection in a non-human primate model, the immunosuppressive agents used for allogeneic cardiac transplantation in the clinical setting were insufficient for the graft to survive for more than 6 months even in the MHC-matching transplantation [16]. Co-transplantation of other cells that had shown immunosuppressive effects through Tregs, such as vasculogenically conditioned PBMCs or mesenchymal stem cells, reportedly prolonged graft survival after allogeneic iPSC-CM transplantation in mice [17, 18]. However, the engraftment of the transplanted cells was hardly observed 2 weeks after allogeneic iPSC-CM transplantation, shown in studies using vasculogenically conditioned PBMCs and mesenchymal stem cells [17, 18].

Immunosuppressants in clinical use suppress T-cell activation and B-cell differentiation by inhibiting the transcription of IL-2 or downstream signaling of the IL-2 receptor or suppressing the proliferation of lymphocytes by inhibiting DNA synthesis, which leads to nonspecific immunosuppression rather than donor-specific immunosuppression [19–21]. Nonspecific immunosuppression might cause side effects such as susceptibility to infection and malignant tumors [22–25]. However, the mixed-chimeric mice established herein showed a donor-specific immunosuppressive effect, which would have a lower risk of infection and malignancy compared to a nonspecific one [12].

These mixed-chimera mice were established using RGI-2001 and anti-CD154 antibodies in an irradiated murine bone marrow transplant model. RGI-2001 activated iNKT cells, which are innate lymphocytes that produce a variety of cytokines and bridge the innate and adaptive immune systems [26] and induce the proliferation of CD4^+^CD25^+^Foxp3^-^ preTregs, which differentiate into CD4^+^CD25^+^Foxp3^+^ Tregs upon IL-2 stimulation [27]. This is consistent with previous reports stating that the use of a calcineurin inhibitor, which inhibits IL-2 transcription, failed to induce Treg expansion [28].

The expansion of Tregs and donor-derived dendritic cells in the recipient thymus was accompanied by a sequential clonal deletion in the donor-reactive CD8^+^ T cells secreting IFN-g, which led to the engraftment of donor bone marrow cells [13]. The transplantation of donor splenocytes instead of bone marrow cells fails to expand Tregs, depletes donor-reactive CD8^+^ T cells, and prolong graft survival [29]. This indicates that donor bone marrow cells are required for further expansion of Tregs and are indispensable for the establishment of mixed-chimera mice. Although the co-transplantation of donor splenic T cells reduced the number of bone marrow cells down to one-fourth, the requirement of harvesting donor bone morrow cells makes it difficult to apply this model for human allogeneic iPSC-CMs transplantation [30]. The identification of the responsible cell type in bone marrow cells for the establishment of mixed-chimera mice and its differentiation from iPSCs could help induce mixed-chimera mice without transplantation of donor bone marrow cells, which would be clinically acceptable. This method would be applicable for allogeneic iPSC-CMs, as well as all other allogeneic iPSC-based regenerative therapies, inducing donor-specific immunotolerance.

In this study, the allogeneic iPSC-CM sheets were transplanted into the subcutaneous space of non-ischemic mice instead of onto the heart of disease model, which is one of the limitations of this study because immune reactions may be altered by morbidities or different transplantation sites. Since undifferentiated cells in iPSC-CMs were not eliminated herein, transplanted cells induced tumorigenesis, resulting in teratomas formation, which was supported by the previous study [31]. Undifferentiated cells in human iPSC-CMs were previously completely eliminated, such that human iPSC-CMs transplanted in NOD/Shi-scid IL2Rγnull mice did not result in tumorigenesis [31]. Therefore, further studies using complete elimination of undifferentiated cells prior to transplantation would be required to definitively demonstrate the donor-specific immunotolerance in this mixed chimerism.

## Conclusions

In conclusion, mixed chimerism, induced via iNKT cell activation with RGI-2001, prolonged graft survival after allogeneic iPSC-CM transplantation. This donor-specific immunotolerance might potentially shed light on the immune rejection problem after allogeneic iPSC-based therapy.

## Supporting information

S1 Data (XLSX)
